# The relationships between hemoglobin and insulin resistance, glucose effectiveness, and first- and second-phase insulin secretion in adult Chinese

**DOI:** 10.20945/2359-3997000000169

**Published:** 2019-08-28

**Authors:** Yen-Shan Yang, Chung-Ze Wu, Jiunn-Diann Lin, Chang-Hsun Hsieh, Yen-Lin Chen, Dee Pei, Shi-Wen Kuo

**Affiliations:** 1 Department of Medicine School of Medicine Fu-Jen Catholic University New Taipei City Taiwan Department of Medicine , School of Medicine , Fu-Jen Catholic University , New Taipei City , Taiwan; 2 Department of Internal Medicine Shuang Ho Hospital Taipei Medical University Taiwan Division of Endocrinology, Department of Internal Medicine, Shuang Ho Hospital, Taipei Medical University; Division of Endocrinology and Metabolism, Department of Internal Medicine, School of Medicine, College of Medicine, Taipei Medical University, Taipei, Taiwan; School of Medicine College of Medicine Taipei Medical University Taipei Taiwan; 3 Division of Endocrinology and Metabolism Department of Internal Medicine Tri-Service General Hospital Taipei Taiwan Division of Endocrinology and Metabolism , Department of Internal Medicine , Tri-Service General Hospital , Taipei , Taiwan; 4 Cardinal Tien Hospital School of Medicine Fu-Jen Catholic University New Taipei City Taiwan Department of Pathology, Cardinal Tien Hospital , School of Medicine , Fu-Jen Catholic University , New Taipei City , Taiwan; 5 Catholic Fu-Jen Hospital School of Medicine Fu-Jen Catholic University New Taipei City Taiwan Department of Internal Medicine, Catholic Fu-Jen Hospital , School of Medicine , Fu-Jen Catholic University , New Taipei City , Taiwan; 6 Department of Endocrinology Taipei Tzu Chi Hospital Buddhist Tzu Chi Medical Foundation Taiwan Department of Endocrinology , Taipei Tzu Chi Hospital , Buddhist Tzu Chi Medical Foundation , Taiwan

**Keywords:** Diabetes mellitus, hemoglobin, first phase insulin secretion, second phase insulin secretion, glucose effectiveness, insulin resistance

## Abstract

**Objective:**

We denote the four major factors related to the development of type 2 diabetes (T2D) as “diabetes factor” (DF); increased insulin resistance (IR); decreased glucose effectiveness (GE); and the first-and-second-phase of insulin secretion (FPIS, SPIS). The level of hemoglobin (Hb) was found to be related to IR and FPIS, but no-known studies focused on its role in relation to SPIS and GE. In this study, we aim to evaluate the relationships between Hb and all four DFs in the same individual.

**Subjects and methods:**

We randomly enrolled 24,407 men and 24,889 women between 30 and 59 years old. IR, FPIS, SPIS and GE were measured according to equations published in our previous studies. To compare the slopes between Hb and the four DFs with different units, we converted their units to percent of change per unit of increased Hb.

**Results:**

Age, HDL-cholesterol and GE were higher in women; BMI, blood pressure, LDL-cholesterol, TG, Hb, FPIS, SPIS and IR were higher in men. After they were converted into percentage, the closeness of their relationships to Hb, from the highest to the lowest, were GE, IR, FPIS and SPIS for women and IR, GE, FPIS and SPIS for men. GE was the only one negatively related to Hb.

**Conclusions:**

Our data showed that IR, FPIS and SPIS were both positively and, GE negatively, related to the Hb in adult Chinese. For women, GE had the closest association with Hb; for men, it was IR. Both phases of insulin secretion had relatively weaker relationships than IR and GE.

## INTRODUCTION

In the past two decades, the prevalence of obesity has increased dramatically in Taiwan as well as globally ( 1 ). One of the possible contributors is a more westernized lifestyle. Alongside this phenomenon the incidence of type 2 diabetes (T2D) also parallels the obesity issue ( 2 ). Nowadays, it is estimated that its prevalence has reached 6.38% (in ages 20-79) ( 3 ). This untoward change is a burden not only to the individual but also to the nation’s health providers and government. Regrettably, until now, the underlying pathophysiology of T2D is still under investigation. Obviously, there might be different “sub-types” of diabetes ( 4 , 5 ). What is known is that there are four factors which all contribute to the development of diabetes (diabetes factor; DF), i.e., increased insulin resistance (IR), decreased insulin secretion both first- and second-phase (FPIS, SPIS, respectively), and decreased glucose effectiveness (GE). It is interesting to note that both IR and insulin secretion have been studied extensively in the past ( 6 - 9 ). However, the roles of different phases of insulin secretion and GE were much less understood. This is probably due to the complexity and high cost of acquiring the measurements of these three factors ( 10 ).

As early as 2003, Choi and cols. were the first to propose that hemoglobin (Hb) is positively related to IR ( 11 ). The relationship could be easily explained by the stimulation of insulin to the growth of human erythroid progenitors in vitro ( 12 ). Other than IR, by using oral glucose tolerance tests, FPIS has also been reported to relate to Hb ( 13 ). However, to our knowledge, there has been no study focusing on the relationships between Hb, SPIS and GE.

In the present study, we enrolled 49.295 adults to evaluate the relationships between Hb and all four DFs in the same individual. Thus, we can further understand the role of Hb in the pathophysiology of T2DM.

## SUBJECTS AND METHODS

### Study subjects

We randomly enrolled 24,407 men and 24,489 women aged between 30 and 59 years old from an MJ Health Screening Center in Taiwan in 2011 and 2012. They underwent routine health checks at the time of the study. MJ Health Screening Centers are private chain-clinics located throughout Taiwan. They provide regular health examinations for their members. All study participants were anonymous and gave informed consent. Data were provided by MJ Health Screening Centers for research purposes only, and the institutional review board of MJ Health Screening Center approved the study protocol. Participants who were on any medications known to affect blood pressure, glucose and lipids levels were excluded. They were further divided into two groups: those with metabolic syndrome (MetS), and those without, according to the World Health Organization criteria ( 14 ). In order to observe the effect of Hb, they were further grouped into four groups according to the quartiles of Hb levels.

On the day of the study, senior nursing staff obtained subjects’ medical history, including information on current medications via questionnaire, followed by a complete physical examination. Waist circumference (WC) was measured horizontally at the level of the natural waist, which was identified as the level at the hollow molding of the trunk when the trunk is laterally concave. BMI was calculated as the subject’s body weight (kg) divided by the square of the subject’s height (m). Both systolic blood pressure (SBP) and diastolic blood pressure (DBP) were measured by nursing staff using standard mercury sphygmomanometers on the right arm of each subject when seated. After the subject had fasted for 10 hours, blood samples were drawn from the antecubital vein for biochemical analysis. The plasma used for analyzing fasting plasma glucose (FPG) and lipid proﬁles was separated from the blood within 1 hour before the experiment. FPG was measured using a glucose oxidase method (YSI 203 glucose analyzer, Yellow Springs Instruments, Yellow Springs, USA). Total cholesterol and triglycerides (TG) were measured using a dry, multilayer analytical slide method with the Fuji Dri-Chem 3000 analyzer (Fuji Photo Film, Tokyo, Japan). Serum high-density lipoprotein cholesterol (HDL-C) and low-density lipoprotein cholesterol (LDL-C) concentration were analyzed using an enzymatic cholesterol assay following dextran sulfate precipitation.

In order to quantify the DFs, we used the equations taken from our study groups and these are listed below (in international units). To demonstrate the reliability of our equations, a short statement is given here. When performing these studies data, approximately 70% of the participants were used to build the equation while data from the remaining 30% were used for external validation. Thus, the accuracy of the equations could be tested.

1. IR: In total, there were 327 subjects enrolled. The IR was measured by insulin suppression test. The r value between the measured and calculated GE was 0.581 ( *p* < 0.001). It was published in “Journal of Diabetes Investigation” in 2013.

2. IR = log (1.439 + 0.018 × sex - 0.003 × age + 0.029 × BMI - 0.001 × SBP + 0.006 × DBP + 0.049 × TG - 0.046 × HDLC - 0.0116 × FPG) × 10 ^3.333^ ( 15 ).

3. FPIS: In total, there were 186 subjects enrolled. The FPIS was measured by frequent sampled intravenous glucose tolerance tests. The r value between the measured and calculated GE was 0.671 ( *p* < 0.000).

FPIS = 10 ^(1.477 - 0.119 × FPG + 0.079 × BMI - 0.523 × HDLC)^ ( 16 ).

SPIS: In total, there were 82 participants. The SPIS was measured by a modified low dose glucose infusion test. The r value between the measured and calculated GE was 0.65 ( *p* = 0.002). SPIS = 10 ^(-2.4 - 0.088 × FPG + 0.072 × BMI)^ ( 17 ).

4. GE: In total, there were 227 participants. The GE was measured by frequent sampled intravenous glucose tolerance tests. The r value between the measured and calculated GE was 0.43 ( *p* = 0.001).

GE = (29.196 - 0.103 × age - 2.722 × TG - 0.592 × FPG) ×10 ^-3^ ( 18 )

### Statistical analysis

All statistical analyses were performed using SPSS 19.0 (IBM Inc., Armonk, New York). Data are presented as mean ± standard deviation. All data were tested for normal distribution with the Kolmogorov-Smirnov test and for homogeneity of variances with Levene’s test. The data of FPIS, SPIS and TG were log transformed before analysis due to the fact that they were not normally distributed. The *t* – test was performed to evaluate the differences between normal and diabetic groups. To evaluate the differences of the mean values of the four groups, from the highest to the lowest levels of Hb, one-way analysis of variance was used. Bonferroni was chosen as the post hoc method for comparison between groups.

**Figure 1:**
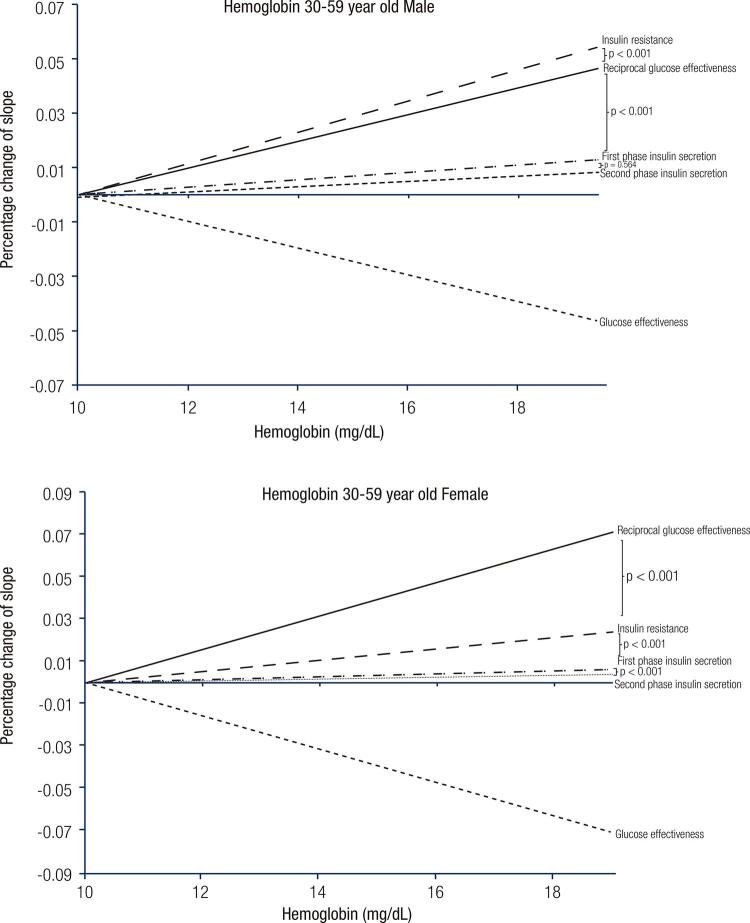
The comparison of the slopes between hemoglobin and diabetes factors in males and females.

Simple correlation was applied to evaluate the relationships of two independent variables such as Hb and IR. Simultaneously, the slopes of these relationships could also be obtained. Since the units and scales were different for these four lines, it was impossible to compare their slopes, which represent the rate changes of each factor when the Hb level is higher. In order to solve this problem, rather than plotting each factor against the level of Hb with the original units, we used uniquely designed lines to compare their slopes. Take FPIS as an example, it could be seen clearly since the lowest level of the regression line was 0.20 and the highest was 4668.4. We took the highest value of FPIS as 100% while the lowest was 0%. The other values between these two extremes were calculated into the percentage correspondingly. In other words, the true values of FPIS were divided by 4668.2 and multiplied by 100. Thus, by using this method, different units from different factors were all changed to percentage and could then be compared ( Figure 1 ). Among these four factors, only the GE had a negative correlation while Hb is higher. In order to compare the slope of GE with the other three factors, we plotted a mirror-line (or reciprocal) from the 4 ^th^ quadrant to the 1 ^st^ quadrant but with the same slope.

## RESULTS

Table 1 shows all the demographic data and parameters derived from our equations for men and women. Age, HDL-cholesterol and GE were higher in women and, at the same time, BMI, blood pressure, LDL-cholesterol, TG, Hb, FPIS, SPIS and IR were higher in men. All these differences reached statistical significances. In Table 2 , the participants were divided into four groups according to the Hb quartiles. It is not surprising to find that all these data were higher in subjects with the highest quartile of Hb, except for GE and HDL-cholesterol. Table 3 depicts the results showing simple correlation between Hb and the other four factors. It can be noted that IR correlated most closely with Hb in men (r = 0.210, p < 0.001) and with GE in women. Among the DFs, GE was the only one which was negatively correlated with Hb. However, it should be remembered that these r values should not be compared since they belong to different units.

**Table 1:** Demographic data and diabetes factors of women and men

	Men	Women	*p*
n	24407	24889	
Age (year)	41.5 ± 8.3	42.8 ± 8.7	< 0.001
Body mass index (kg/m ^2^ )	24.1 ± 2.9	23.1 ± 3.2	< 0.001
Systolic blood pressure (mmHg)	118.7 ± 13.8	114.8 ± 15.3	< 0.001
Diastolic blood pressure (mmHg)	75.6 ± 10.2	72.3 ± 12.1	< 0.001
HDL-C (mmol/L)	1.1 ± 0.3	1.3 ± 0.3	< 0.001
LDL-C (mmol/L)	3.4 ± 0.9	3.3 ± 0.8	< 0.001
TG (mmol/L)	1.6 ± 0.9	1.2 ± 0.7	< 0.001
Hemoglobin (10 ^3^ /μL)	15.2 ± 1.0	13.1 ± 1.0	< 0.001
FPIS (μU/min)	197.3 ± 204.612	136.9 ± 171.854	< 0.001
SPIS (pmol/mmol)	0.082 ± 0.065	0.073 ± 0.069	< 0.001
IR (10- ^4^ ∙ min- ^1^ ∙ pmol- ^1^ ∙ L- ^1^ )	3.70 ± 0.025	3.69 ± 0.024	< 0.001
GE (10- ^2^ ∙ dL ∙ min- ^1^ ∙ kg- ^1^ )	0.017 ± 0.003	0.018 ± 0.002	< 0.001

FPIS: first phase insulin secretion; SPIS: second phase insulin secretion; IR: insulin resistance; GE: glucose effectiveness; HDL-C: high-density lipoprotein cholesterol; LDL-C: Low-density lipoprotein cholesterol; TG: Triglyceride, Data are shown mean ± SD.

**Table 2:** Demographic data and diabetes factors in different hemoglobin quartiles

	Hemoglobin 1			Hemoglobin 2			Hemoglobin 3			Hemoglobin 4			Total			p

Men																
n	6490			5857			5843			6216			24406			
Age (year)	43.1 ± 8.7 ^234^			41.6 ± 8.2 ^14^			41.1 ± 8.1 ^14^			40.3 ± 7.9 ^123^			41.5 ± 8.3			< 0.001
Body mass index (kg/m ^2^ )	23.6 ± 2.8 ^234^			24.0 ± 2.9 ^134^			24.2 ± 2.9 ^124^			24.6 ± 3.0 ^123^			24.1 ± 2.9			< 0.001
Systolic blood pressure (mmHg)	117.7 ± 14.1 ^34^			118.4 ± 13.6 ^4^			118.9 ± 13.7 ^1^			119.9 ± 13.6 ^12^			118.7 ± 13.8			< 0.001
Diastolic blood pressure (mmHg)	74.0 ± 10.1 ^234^			75.3 ± 9.9 ^14^			75.8 ± 10.1 ^14^			77.5 ± 10.3 ^123^			75.6 ± 10.2			< 0.001
HDL-C (mmol/L)	1.091 ± 0.320			1.091 ± 0.319			1.084 ± 0.319			1.075 ± 0.326			1.085 ± 0.321			0.018
LDL-C (mmol/L)	3.313 ± 0.843 ^234^			3.421 ± 0.844 ^1^			3.455 ± 0.865 ^1^			3.479 ± 0.875 ^1^			3.415 ± 0.859			< 0.001
TG (mmol/L)	1.418 ± 0.812 ^234^			1.545 ± 0.870 ^134^			1.622 ± 0.912 ^124^			1.769 ± 0.942 ^123^			1.587 ± 0.894			< 0.001
Hemoglobin (10 ^3^ /μL)	14.0 ± 0.7 ^234^			15.0 ± 0.2 ^134^			15.5 ± 0.2 ^124^			16.4 ± 0.5 ^123^			15.2 ± 1.0			< 0.001
FPIS (μU/min)	178.526 ± 196.749 ^23^			191.953 ± 198.036 ^4^			200.705 ± 192.282 ^14^			218.908 ± 226.726 ^123^			197.345 ± 204.614			< 0.001
SPIS (pmol/mmol)	0.076 ± 0.076 ^23^			0.081 ± 0.060 ^4^			0.083 ± 0.062 ^14^			0.088 ± 0.061 ^123^			0.082 ± 0.065			< 0.001
IR (10 ^-4^ ∙ min ^-1^ ∙ pmol ^-1^ ∙ L ^-1^ )	3.693 ± 0.024 ^234^			3.698 ± 0.024 ^134^			3.701 ± 0.025 ^124^			3.706 ± 0.025 ^123^			3.700 ± 0.025			< 0.001
GE (10 ^-2^ ∙ dL ∙ min ^-1^ ∙ kg ^-1^ )	0.018 ± 0.003 ^23^			0.017 ± 0.003 ^4^			0.017 ± 0.003 ^14^			0.017 ± 0.003 ^123^			0.017 ± 0.003			< 0.001

**Women**																

n	5808			5858			6537			6686			24889			
Age (year)	42.1 ± 8.1 ^34^			42.5 ± 8.6 ^4^			42.8 ± 8.7 ^14^			43.8 ± 8.9 ^123^			42.8 ± 8.7			< 0.001
Body mass index (kg/m ^2^ )	22.6 ± 2.9 ^234^			22.9 ± 3.0 ^134^			23.1 ± 3.2 ^124^			23.6 ± 3.4 ^123^			23.1 ± 3.2			< 0.001
Systolic blood pressure (mmHg)	112.8 ± 14.8 ^34^			113.4 ± 14.8 ^34^			114.9 ± 15.2 ^124^			117.8 ± 15.7 ^123^			114.8 ± 15.3			< 0.001
Diastolic blood pressure (mmHg)	70.5 ± 14.3 ^34^			71.1 ± 9.9 ^34^			72.7 ± 10.0 ^124^			74.7 ± 13.1 ^123^			72.3 ± 12.1			< 0.001
HDL-C (mmol/L)	1.287 ± 0.344			1.297 ± 0.345			1.300 ± 0.342			1.305 ± 0.352			1.297 ± 0.346			0.046
LDL-C (mmol/L)	3.121 ± 0.807 ^234^			3.221 ± 0.808 ^134^			3.291 ± 0.803 ^124^			3.424 ± 0.867 ^123^			3.271 ± 0.830			< 0.001
TG (mmol/L)	1.039 ± 0.572 ^234^			1.103 ± 0.607 ^134^			1.168 ± 0.659 ^124^			1.310 ± 0.748 ^123^			1.161 ± 0.662			< 0.001
Hemoglobin (10 ^3^ /μL)	11.8 ± 0.6 ^234^			12.8 ± 0.2 ^134^			13.3 ± 0.2 ^124^			14.2 ± 0.5 ^123^			13.1 ± 1.0			< 0.001
FPIS (μU/min)	128.189 ± 166.258 ^4^			132.475 ± 167.439 ^4^			137.168 ± 153.631			148.042 ± 195.177 ^12^			136.888 ± 171.854			< 0.001
SPIS (pmol/mmol)	0.068 ± 0.059 ^34^			0.070 ± 0.055 ^4^			0.074 ± 0.071 ^14^			0.079 ± 0.085 ^123^			0.073 ± 0.069			< 0.001
IR (10 ^-4^ ∙ min ^-1^ ∙ pmol ^-1^ ∙ L ^-1^ )	3.684 ± 0.023 ^234^			3.686 ± 0.022 ^134^			3.689 ± 0.024 ^124^			3.693 ± 0.026 ^123^			3.688 ± 0.024			< 0.001
GE (10 ^-2^ ∙ dL ∙ min ^-1^ ∙ kg ^-1^ )	0.019 ± 0.002 ^234^			0.019 ± 0.002 ^134^			0.018 ± 0.002 ^124^			0.018 ± 0.003 ^123^			0.018 ± 0.002			< 0.001

FPIS: first phase insulin secretion; SPIS: second phase insulin secretion; IR: insulin resistance; GE: glucose effectiveness; HDL-C: high-density lipoprotein cholesterol; LDL-C: Low-density lipoprotein cholesterol; TG: Triglyceride, Data are shown mean ± SD.

**Table 3:** Simple correlation between hemoglobin and diabetes factors

			r		P	

Men						
First phase insulin secretion			0.079		< 0.001	
Second phase insulin secretion			0.077		< 0.001	
Insulin resistance			0.210		< 0.001	
Glucose effectiveness			-0.105		< 0.001	

**Women**						

First phase insulin secretion			0.043		< 0.001	
Second phase insulin secretion			0.061		< 0.001	
Insulin resistance			0.154		< 0.001	
Glucose effectiveness			-0.182		< 0.001	

Figure 1 is the most important finding in the present study and it demonstrates the slopes of the four factors. Again, as stated previously in Methods, all four factors were converted to 100% because they had different units and were not comparable. GE was the only factor that needed to be reciprocally plotted. because it was negatively correlated to the level of Hb. It is clear in the figure that they were all significantly related to Hb. However, the closeness of their relationships, from the highest to the lowest, were GE, IR, FPIS and SPIS for women, and IR, GE, FPIS and SPIS for men.

## DISCUSSION

In the present study, we demonstrated that IR, FPIS, and SPIS were all positively, and GE negatively, related to Hb in both genders. Moreover, our data also showed that the order of the closeness, from the highest to the lowest, was IR, GE, FPIS and SPIS for men and GE, IR, FPIS and SPIS for women. Our study is the first to evaluate all the four DFs simultaneously in the same individual. This could help us to understand the role of Hb in diabetes more thoroughly.

Wannamethee and cols. were the first to report that raised hematocrit level, which is a major determinant of whole blood viscosity, is related to increased incidence of T2D ( 19 - 22 ). However, due to a suboptimal definition for diabetes and its selected cohort, it is less persuasive. Following this study, results of the longitudinal Atherosclerosis Risk in Communities (ARIC) study successfully validated this observation ( 20 ). The connection between increased T2D and Hb could be explained by the data of Choi and cols *.* By using a homeostasis model assessment of IR, they showed a positive correlation between Hb and IR. The r value was around 0.1 to 0.2 according to gender and whether they were smoking ( 11 ). As it is well known that increased IR is one of the most important pathophysiology factors for having diabetes, the results of the present study are in line with those mainstream studies. The r value was 0.154 for women and 0.210 for men which is quite close to their finding.

There are two hypotheses underlying the relationship. The first one is the effect of increased blood viscosity, which decreases microvascular blood flow and causes inadequate delivery of insulin to multiple tissues. Thus, blood flow-related insulin resistance occurs ( 21 ). The second one relates to the roles of sex hormones, both androgen and estrogen. For example, post-menopause women who receive estrogen-replacement therapy would have higher IR ( 23 ). As for androgen, data from the prostate cancer patients treated with androgen-deprivation therapy indicated that after chemical castration, the positive influence from the androgen disappeared and IR increased ( 24 ). On the contrary, the role of androgen in patients with polycystic ovary syndrome is different. Hyperandrogenism is connected to IR in those patients. In addition, it is well-known that androgen is strongly related to erythrocytosis, which causes men to have a higher Hb level than women ( 25 ). Based on the aforementioned discussion, we can concluded that IR might influence the Hb level via the effects of sex hormones. In the present study, we further confirm the relationship between Hb and IR. However, the average r value between Hb and IR is lower in the Korean study (0.16) ( 11 ). This could be explained by several differences in the conditions between studies such as age, BMI and ethnicity.

There have been very few studies focused on the relationships between Hb and insulin secretion. Until now, results from different studies are still held in controversy. In the present study, our data suggests that subjects with higher Hb levels had higher insulin secretion, both first and second-phase. Other researchers concluded similarly. For example, the aforementioned report by Facchini and cols. not only found a positive correlation between Hb and IR, but also a compensatory increase of the insulin secretion for the deterioration of IR ( 26 ). This association is not entirely surprising since body weight has long been confirmed to be positively correlated with Hb ( 27 , 28 ). At the same time, higher body weight could be translated to more insulin secretion ( 29 , 30 ). Thus, the role of body weight is like a bridge between Hb and insulin secretion. On the contrary, Shimodaira and cols. showed the opposite result i.e., that there was a negative relationship between Hb and insulin secretion. However, this relationship is only significant for men (r = -0.197 for men and -0.082 for women) ( 13 ). To explain this, they suggested that oxidative stress might be the cause of lower insulin secretion ( 31 ). Similar to the relationship between Hb and IR, the discrepancy found in these studies might be due to age, BMI, ethnicity, or the methods used to measure insulin secretion. Further studies are needed to elucidate this fascinating and complex interaction.

As mentioned in the introduction, given the important role that GE might have, its relationship with GE has never been studied. Our study is the first to demonstrate that there is a negative association between Hb and GE. The mechanism between them remains obscure and could be discussed from several aspects with the hypothesis that obesity plays a central role. These chain-reactions start from obesity. Evidence suggests that obesity could trigger erythropoietin production and thus stimulate synthesis of Hb ( 32 , 33 ). At the same time, by using an oral glucose tolerance test derived from GE, Weiss and cols. published a study in Diabetes Care in 2015 ( 34 ). A strong negative association was reported between GE and waist circumference (r = -0.67). Given the large n number in that study, this result should be reliable. Data from other studies also supported their finding ( 22 ). To put it simply, obesity can induce higher Hb concentration and, at the same time, a lower GE level. However, this hypothesis still needs further studies for full verification.

Even though we believe that our study provides novel information, there are still limitations. First, this is only a cross-sectional study. Compared to the longitudinal study, it provides less solid evidence. Second, the methods we used are likely to be less accurate than more sophisticated tests such as the intravenous glucose tolerance test or clamp. However, the substantial body of fellow research students might remedy this issue. Third, this study is applied to a homogenous ethnic group. Caution should be exercised when extrapolating our findings to other ethnic groups. Finally, the controversial relationships between Hb and insulin secretion have still not been solved. Further basic or clinical studies are needed to support our findings.

In conclusion, our data showed that IR, FPIS and SPIS were positively, and GE negatively, related to the Hb in adult Chinese. In women, GE had the closest association with Hb; in men, it is IR. Both phases of insulin secretion had relatively weaker relationships than IR and GE.
